# Spontaneous Regression of Primary Thyroid Lymphoma: Two Rarities at the Same Time

**DOI:** 10.5146/tjpath.2018.01445

**Published:** 2020-05-15

**Authors:** Francisco Illan-Gambin, Pablo Manresa-Manresa, Estefanía Rodriguez-Aleo, Ignacio Aranda-Lopez

**Affiliations:** Department of Pathology, University General Hospital of Alicante, Alicante, Spain; Department of Hematology, University General Hospital of Alicante, Alicante, Spain; Nursing Department, University General Hospital of Alicante, Alicante, Spain

**Keywords:** Lymphoma, Thyroid neoplasms, Spontaneous neoplasm regression, PD-L1

## Abstract

Primary thyroid lymphomas are pretty uncommon, and constitute about 5% of the neoplasms of this organ. Spontaneous tumor regression is defined as the total or partial disappearance of a tumor as proven by microscope without treatment or under inadequate treatment. It is estimated to happen in one out of 60,000-100,000 cases. We present a case of primary thyroid lymphoma with spontaneous regression after diagnostic puncture and corroborated with hemithyroidectomy at four months. The patient died after twenty-six months of follow-up because of endocarditis and there was no relapse at any time.

## INTRODUCTION

Primary thyroid lymphomas constitute about 5% of all primary thyroid neoplasms and around 2% of extranodal lymphomas. The prototype patient is a woman (up to 4 times more frequent than in men) around the sixth decade of life. As regards histological subtypes, diffuse large B-cell lymphoma and marginal zone lymphoma are the most common (1). Spontaneous tumor regression (STR) is defined as the total or partial disappearance of a tumor validated microscopically without treatment or under inadequate treatment. It is estimated to happen in one out of 60,000-100,000 cases, although it probably appears more often. It is difficult to study STR because of its low prevalence. In addition, regressive changes are usually does not reported when they are presented in a partial way (2). Among solid tumors, melanoma seems to have STR most frequently. Regarding lymphomas, low grade types are more likely to regress but it is exceptional in those of high grade (3).

## CASE REPORT

**Figure 1:**
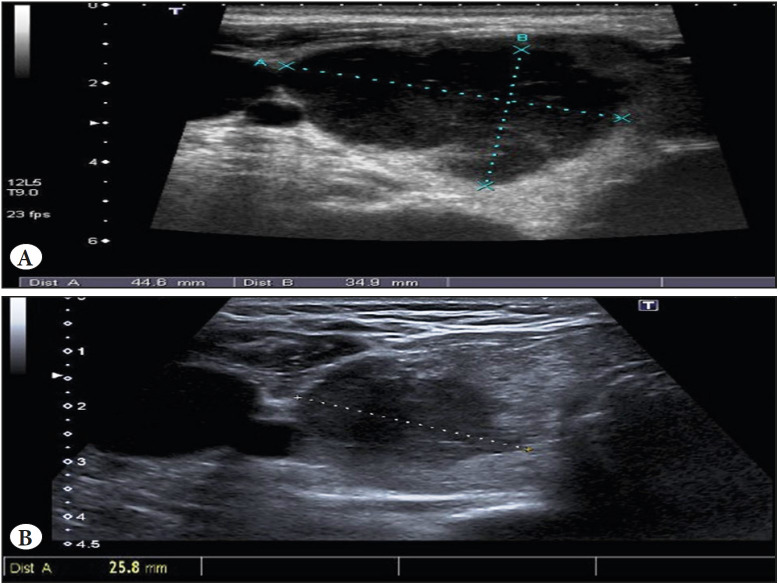
Right thyroid ultrasonography. The tests were performed two months apart. (upper: first ultrasonography). Note the tumor measures.

A 63-year-old woman had been followed-up for the last year because of subclinical hypothyroidism. She had been surgically treated for tonsils in childhood and for her gallbladder due to stones, and for aortic-mitral double replacement 20 years ago secondary to endocarditis. Because of the last one, she was anticoagulated with warfarin. Suddenly, she noted a nodule in the front of the neck that became visible, without any other symptoms. The ultrasound showed a right thyroid lobe replaced by a solid and heterogeneous tumor of 4.4 x 3.4 cm with ill-defined contours and abnormal vascularization with Doppler (Figure 1). The patient underwent an extraclinical fine-needle aspiration (FNA) which displayed lymphocytes and large blastic-type cellularity with dispersed epithelial elements suggestive of thyroiditis but lymphoproliferative disorder could not be ruled out. Then she was referred to our hospital for a needle biopsy (16G).

**Figure 2:**
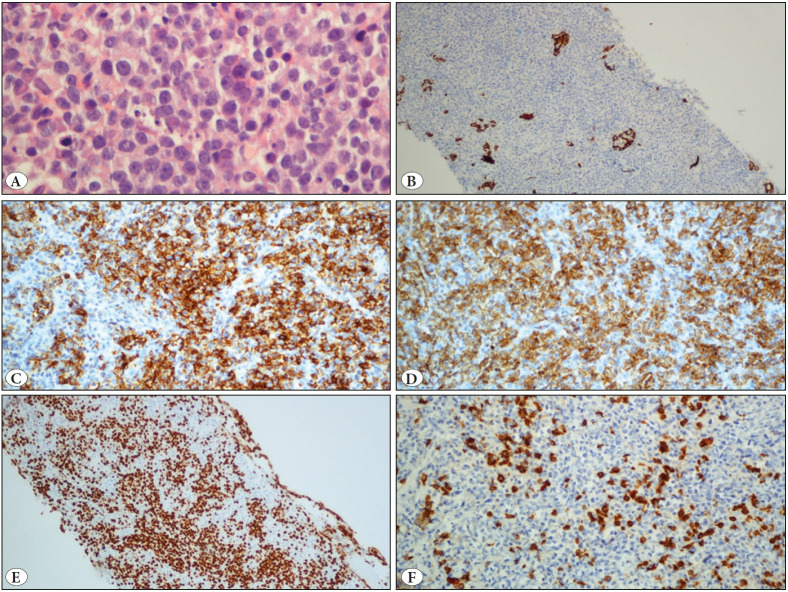
Primary thyroid diffuse large B-cell lymphoma. **A)** Lymphoid large cells with lobulated nuclei, marked nucleoli, frequent mitosis and apoptosis are present (H&E; x 600). **B)** Nests of thyroid epithelium are identified (CKAE1/AE3; x100). **C)** Cells show positivity for CD20 (IHC; x200). **D)** Cells show positivity for CD10 (IHC; x200). **E)** High proliferation (Ki67; x100). **F)** There is accompanying T-lymphocytes (CD3; x200).

The tissue showed a diffuse infiltrate of lymphoid large cells with lobulated nuclei, marked nucleoli, frequent mitosis and apoptosis. With broad-spectrum cytokeratins (CK AE1-AE3), nests of dispersed thyroid epithelium were identified. After immunohistochemical evaluation, the lymphoid nature of the neoplasm (CD45 +) was confirmed. It showed germinal center lymphocyte B-cell differentiation (CD20 +, CD10 +, BCL-2 +, MUM1 +, BCL-6 - and c-MYC -) and it had accompanying T lymphocytes (CD3 +). The proliferation index with Ki67 was greater than 80% (Figure 2) and the immunoglobulin chain rearrangement analysis showed IgH clonality. Staining for PD-L1 (clone 22C3) showed low expression in atypical lymphoid cells (1-2%). As there was no adenopathy or alterations on the imaging tests (including PET/CT), the diagnosis of primary thyroid diffuse large B-cell lymphoma, germinal center B-cell subtype (WHO, 2017) was made.

**Figure 3:**
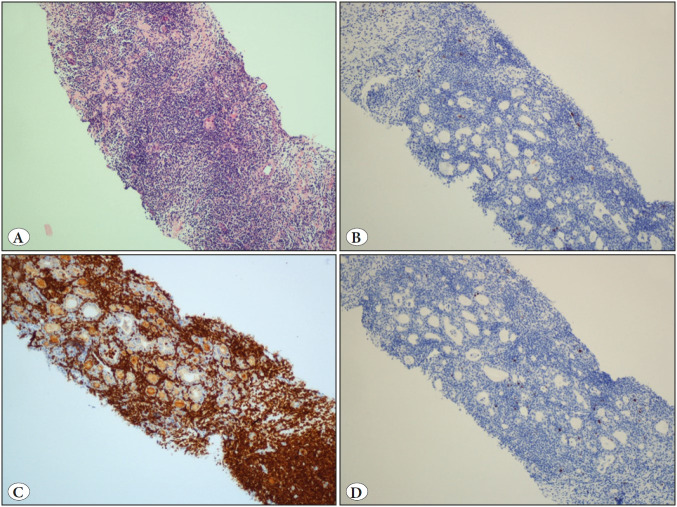
**A)** Thyroid with small lymphocytes infiltration (H&E; x100). **B)** Cells are CD20 negative (IHC; x100). **C)** Cells are CD3 positive (IHC; x100). **D)** Ki67 is less than 1% (IHC; x100).

At the time of staging, two months after the first FNA, a clinical decrease of tumor dimensions was observed so a new ultrasound was performed. It showed a nodule with the same characteristics as the previous one that now measured 2.5 cm (Figure 1). A new biopsy was taken showing thyroid follicles, fibrous tracts and non-atypical small lymphocytes. There was also plasma cells and macrophages with no apparent neoplastic population. The absence of tumor cells was confirmed immunohistochemically (CD20 -, CYCLIN D1 -, CD5 - and CD10 -) with Ki67 less than 1% and a marked increase in the T-cell population (Figure 3). Residual lymphocytes showed no morphological or immunohistochemical characteristics suggestive of any other type of lymphoma.

**Figure 4:**
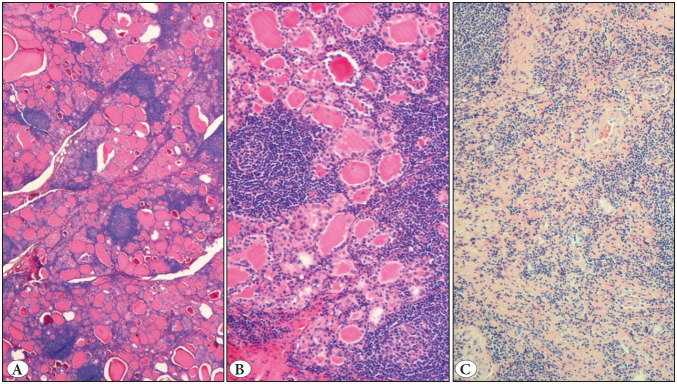
**A-B)** Thyroid showing features of thyroiditis with follicles of variable sizes and lymphoid clusters forming germinal centers (H&E; x20, H&E; x200). **C)** In some areas, fibrotic bands with lymphocytes are present (H&E; x100).

Finally, a hemithyroidectomy was carried out five months after the first FNA. The right hemithyroid had regular weight and dimensions and whitish fibrous bands without defined solid nodules. After microscopic examination, we showed thyroid follicles of variable sizes with lymphoid clusters and germinal centers as well as a fibrotic area with lymphocytes like scar tissue (Figure 4). No neoplastic population was demonstrated in any slide and the genetic rearrangement did not indicate clonality.

The patient passed away twenty-six months after the diagnosis due to endocarditis and there was no clinical or radiological relapse. In addition, she had undergone a PET/CT scan prior to her death which only showed the infectious cardiac focus without uptake elsewhere. No new treatments were added at any time.

## DISCUSSION

The first cause of lymphoid infiltrate in the thyroid is autoimmune thyroiditis. Currently, it has become the most frequent autoimmune disease and the first cause of hypothyroidism (4). Microscopically it displays lymphocytic infiltration of the stroma with large germinal centers and thyroid follicles. The last ones can show variable degrees of activity with regenerative, oncocytic or atrophic changes (5). Among its complications are lympho- and myeloproliferative disorders, both systemic and primary in the gland, whose relative risk will increase 80 times (6) as well as papillary carcinomas (7). Regarding lymphomas developing on thyroiditis, the most frequent type is marginal zone. It shows a heterogeneous infiltrate with atypical small lymphocytes, centrocytes, immunoblasts and plasma cells that can colonize germinal centers (1).

Meanwhile, STR is a striking entity described by TC Everson and WH Cole in the second half of the 20th century. In their work the definition of STR was proposed for the first time as well as a review of cases published until then (8). Most hypotheses trying to explain it have been in agreement afterwards. Most advocate a massive release of heteroantigens after aggression on the tumor that would enhance the immune response against it. In this way, local or systemic infections, vaccination, contusion, biopsy and even exposure to X-radiation could be triggers. Therefore, the most accepted theory to explain STR attributes is that the immune system plays the main role (9,10). In the current case, we believe that FNA started the T-lymphocyte response that we found on needle biopsy and the surgical specimen and resulted in complete STR.

With reference to non-Hodgkin’s lymphomas, some studies estimate 10-20% of STR in low-grade types but it is anecdotal in high-grade lymphomas. Immunoregulation has been considered important because regression of the lymphoma appears to a greater or lesser degree if T-mediated response is promoted on animal or in vitro models (after virus infection, addition of anti-idiotypic antibodies, cytokines, etc.) (11). There is also an association between the immunity status and the presence of some lymphoproliferative disorders as in AIDS patients, post-transplant lymphoproliferative disorders, and Epstein-Barr virus-associated lymphomas (11,12).

Furthermore, it is known that thyroid lymphomas often show IgH clonality, especially in those from lymphocytic thyroiditis (1), although its prognostic role is unknown nowadays. We found only one published case of thyroid lymphoma regression in which immunoglobulin chain restriction was studied. This patient had IgH clonality and recurrence was earlier (13) as opposed to our case.

Finally, regarding PD-L1, its expression in lymphomas seems to inhibit T-cell activity against the tumor (14). In the present case, the low expression of PD-L1 could be a favorable point for the immune response against the neoplastic population.

## Conflict of Interest

The authors declare no conflict of interest.

## FUNDING

The authors have not received funding from any organization.
